# Epigenomic Transcriptome Regulation of Growth and Development and Stress Response in Cucurbitaceae Plants: The Role of RNA Methylation

**DOI:** 10.3390/cimb47110938

**Published:** 2025-11-11

**Authors:** Guangchao Yu, Zhipeng Wang, Lian Jia, Hua Huang

**Affiliations:** 1 College of Chemistry and Life Sciences, Anshan Normal University, Anshan 114007, China; wangzp1326@gmail.com (Z.W.); jl_58@163.com (L.J.); www.huanghua@163.com (H.H.); 2 Liaoning Key Laboratory of Development and Utilization for Natural Products Active Molecules, Anshan Normal University, Anshan 114007, China

**Keywords:** RNA methylation, Cucurbitaceae, m^6^A, m^5^C, MeRIP-seq, RNA-BisSeq, nanopore direct RNA sequencing, CRISPR/Cas9

## Abstract

RNA methylation, particularly N6-methyladenosine (m^6^A) and 5-methylcytosine (m^5^C), functions as a pivotal post-transcriptional regulatory mechanism and plays a central role in plant growth, development, and stress responses. This review provides a systematic summary of recent advances in RNA methylation research in cucurbit crops. To date, high-throughput technologies such as MeRIP-seq and nanopore direct RNA sequencing have enabled the preliminary construction of RNA methylation landscapes in cucurbit species, revealing their potential regulatory roles in key agronomic traits, including fruit development, responses to biotic and abiotic stresses, and disease resistance. Nevertheless, this field remains in its early stages for cucurbit crops and faces several major challenges: First, mechanistic understanding is still limited, with insufficient knowledge regarding the composition and biological functions of the core protein families involved in methylation dynamics—namely, “writers,” “erasers,” and “readers.” Second, functional validation remains inadequate, as direct evidence linking specific RNA methylation events to downstream gene regulation and phenotypic outcomes is largely lacking. Third, resources are scarce; compared to model species such as Arabidopsis thaliana and rice, cucurbit crops possess limited species-specific genetic data and genetic engineering tools (e.g., CRISPR/Cas9-based gene editing systems), which significantly hampers comprehensive functional studies. To overcome these limitations, future research should prioritize the development and application of more sensitive detection methods, integrate multi-omics datasets—including transcriptomic and methylomic profiles—to reconstruct regulatory networks, and conduct rigorous functional assays to establish causal relationships between RNA methylation modifications and phenotypic variation. The ultimate objective is to fully elucidate the biological significance of RNA methylation in cucurbit plants and harness its potential for crop improvement through genetic and biotechnological approaches.

## 1. Introduction

RNA modification is a fundamental level of post-transcriptional regulation that regulates gene expression without altering the primary nucleotide sequence. This emerging field, known as epitranscriptomics, has developed rapidly over the past decade, revealing more than 17 different RNA chemical modifications in organisms [1]. Among them, RNA methylation-particularly N-^6^methyladenosine (m^6^A) and 5-methylcytosine (m^5^C)—has attracted considerable attention due to its general effects on RNA metabolism, including splicing, stability, translation and decay [2,3].

Regulation of RNA methylation is coordinated by three types of proteins: “writer” (methyltransferase), which makes modifications; “Erasers” (demethylases) remove modifications; and “interpreters” (RNA-binding proteins) that interpret this modification, thereby directing the fate of RNA [4]. In plants, these enzymes are involved in a variety of physiological and developmental processes, including photomorphogenesis, flower formation, embryogenesis and stress adaptation [5,6,7,8]. While most functional insights come from model species such as *Arabidopsis thaliana*, growing evidence suggests that RNA methylation also plays a key role in horticultural crops.

To clearly delineate the core concepts addressed in this review and to facilitate a systematic understanding among readers, key terminology is formally defined herein. Epigenomics, epitranscriptomics, and multi-omics integration represent pivotal domains in contemporary biological research, collectively elucidating the intricate mechanisms of gene expression regulation and their influence on biological development. These fields investigate heritable regulatory processes that operate independently of changes in DNA sequence [9]. Epigenomics focuses on genome-wide regulation of DNA accessibility and transcriptional potential, primarily mediated through mechanisms such as DNA methylation and histone modifications [9,10]. Epitranscriptomics extends epigenomic principles to the RNA level, examining post-transcriptional chemical modifications—including m^6^A and m^5^C—that modulate RNA metabolism (e.g., stability, splicing, translation) and ultimately shape gene expression outcomes [9,11]. These modifications do not alter the RNA nucleotide sequence but instead form a dynamic and functionally significant regulatory layer known as the epitranscriptome [11]. Notably, N6-methyladenosine (m^6^A) is the most prevalent internal modification in eukaryotic messenger RNA and plays a central role in regulating gene expression [12,13]. The m^6^A modification pathway is dynamically controlled by a tripartite system comprising writer proteins (e.g., METTL3, METTL14, WTAP), eraser proteins (e.g., FTO, ALKBH5), and reader proteins (e.g., YTHDF1–3, YTHDC1, YTHDC2) [12,13,14]. Multi-omics integration constitutes a systems biology approach that aims to construct comprehensive and causally interpretable models by jointly analyzing data across multiple molecular layers, thereby uncovering the mechanistic continuum from genomic regulation to phenotypic manifestation [15]. This integrative strategy combines diverse omics data types, including genomics, epigenomics, transcriptomics, proteomics, and metabolomics [16,17].

Cucurbitaceae plants are economically important vegetable crops widely utilized in both fresh consumption and food processing. Key species include melon, watermelon, and pumpkin. Owing to their distinctive features in sex determination, fruit development, and stress resistance, they have emerged as model systems for biological research in plant development and crop improvement [18,19,20]. However, the production of cucurbitaceae crops faces many challenges, including abiotic stresses (such as heat, cold, drought, salinity) and biological stresses (such as cucumber powdery mildew, cosporine leaf spot) [21,22], all of which can seriously affect yield and quality. Therefore, a deep understanding of the molecular mechanisms that regulate the development and stress response of cucurbitaceae plants is crucial for improvement through breeding and biotechnological means. The rapid development of plant genome technology in recent years has provided rich molecular tools for the breeding of Cucurbitaceae plants. For example, through genetic transformation and gene editing techniques, researchers have been able to improve the traits of Cucurbitaceae crops more precisely [23]. For example, CRISPR/Cas9 gene editing technology has been successfully applied to cucumbers to create loss-of-function of powdery mildew resistance genes such as *CsaMLO1*, *CsaMLO8*, and *CsaMLO11*, and has been validated, providing a new approach to improving disease resistance in cucumbers [24]. However, despite significant progress in genomics and breeding, many economically important varieties of cucurbitaceae plants, such as melons and pumpkins, remain difficult to operate effectively through *Agrobacterium* standard transformation methods, which limits the wide application of modern biotechnologies such as gene editing in these crops.

Advances in high-throughput sequencing techniques, such as methylated RNA immunoprecipitation sequencing (MeRIPseq), bisulfite RNA sequencing (RNA-BisSeq), and more recently, nanopore direct RNA sequencing, have begun to paint a picture of RNA methylation in cucumbers and related cucurbitaceae [25,26]. These studies have revealed the dynamic changes in methylation during fruit development, under stress conditions, and in plant–pathogen interactions. However, the field is still in its infancy in this crop: mechanical dissection of methylation “writers”, “erasers”, and “readers” in cucumbers is limited, and functional links between site-specific methylation events and agronomic traits have not yet been firmly established.

Given the rapid accumulation of transcriptome modification data and the potential of RNA methylation in precision breeding, a critical synthesis of current knowledge is timely and necessary. This article aims (1) to review the research progress on plant RNA methylation, with a focus on cucurbit plants; (2) compare methylation mechanisms and functional effects among different species to identify conserved and unique features; (3) highlight the technical approaches for detection and quantification in plants; and (4) discuss current knowledge gaps, future research priorities and potential applications in the Cucurbitaceae plant improvement program, seeking to provide a comprehensive framework for exploring epigenetic transcriptome regulation as a new target for horticultural innovation. To ensure methodological rigor and transparency, we implemented a systematic literature search strategy across multiple electronic databases—including PubMed, Web of Science Core Collection, Scopus, and EBSCO—using a tailored search string such as “(RNA methylation OR m^6^A OR m^5^C) AND (detection OR sequencing)”, adapted to the syntax requirements of each platform. The search was restricted to peer-reviewed original research and review articles published in English between 2000 and 2025. Studies focusing exclusively on DNA methylation or lacking accessible full texts were excluded. The final corpus was determined through a structured process involving removal of duplicates, screening of titles and abstracts, and critical assessment of full-text eligibility.

## 2. The Functional Role and Mechanism of RNA Methylation in the Cucurbitaceae

Some studies have used MeRIP-seq to map the m^6^A landscape of cucumbers. For example, a Cucume database containing information on RNA m^5^C and m^6^A methylation in cucumber and pumpkin was developed, combined with grafting experiments and genetic analysis, aiming to advance the application of RNA methylation in Cucurbiaceae crops in RNA mobility and breeding [25]. Furthermore, Ferraz et al. [26] summarized in a review that the m^6^A level is regulated under heat stress, and the transcripts related to the heat shock response and heat tolerance show a higher degree of methylation. N6-methyladenosine (m^6^A), the most abundant mRNA internal modification in eukaryotes, plays a central role in regulating gene expression and influencing RNA metabolism, and its dynamic reversibility enables it to respond to changes in the internal and external environment. Accumulating evidence indicates that m^6^A modification plays a crucial role in regulating key aspects of plant growth and development, including flowering, seed development, and fruit ripening, through the modulation of critical gene expression. For example, during fruit development, m^6^A methylation directly influences fruit elongation, ripening, size, and quality by regulating the expression of genes associated with cell expansion [27]. However, research on cucurbit crops in this field is still in its infancy, and some initial progress has been made (Table 1). m^6^A methylation is a key RNA epigenetic modification in higher plants and plays an important regulatory role in the ripening process of Oriental melon (*Cucumis melo* L. var. makuwa) fruits. Studies have shown that inhibition of m^6^A demethylase activity can delay fruit ripening and reduce endogenous ethylene production and riping-related indicators. There are eight *ALKBH* family members in the melon genome, among which the expression of *CmALKBH8* is regulated by ethylene signaling, and its transient overexpression significantly promotes the expression of ethylene synthesis genes (*CmACS1*, *CmACS2*, *CmACO1*). It suggests that the gene may be involved in fruit ripening by regulating the m^6^A methylation levels of ethylene synthesis-related genes. These results reveal the molecular mechanism by which m^6^A methylation regulates melon ripening through the ethylene pathway [28]. RNA m^6^A methylation, an important post-transcriptional modification, plays a key regulatory role in watermelons’ response to drought stress. The study identified for the first time watermelon m^6^A methyltransferase *ClMTB*, whose expression is induced by drought stress. Overexpression of *ClMTB* in tobacco can significantly improve drought tolerance in plants by enhancing reactive oxygen species neutralization capacity, alleviating photosynthetic inhibition, and activating multiple hormone and stress response genes. The study revealed that CIMTB-mediated m^6^A modification is a positive regulator of drought adaptation in watermelons, providing new insights into m^6^A-mediated signaling pathways in plant stress [29]. Research has demonstrated that the two RNA methylation modifications, m^6^A and m^5^C, directly regulate the mobility of the key pumpkin gene *CmoCK1* mRNA under low-temperature stress, thereby enhancing plant cold tolerance. This finding indicates that RNA methylation functions not only as a regulator of gene expression but also as a critical molecular switch governing the long-distance transport of mRNA across species in response to environmental stresses [30].

## 3. Advances in Techniques and Methods for RNA Methylation Research and Their Application Prospects in Cucurbitaceae Crops

### 3.1. MeRIP-Seq (m^6^A-Seq)

MeRIP-seq (m^6^A-seq) is a widely used technique for localizing m^6^A modifications in the transcriptome; the core of MerIP-seq lies in immunoprecipitation of RNA fragments containing m^6^A using specific antibodies followed by high-throughput sequencing. This technique has the advantages of high sensitivity, strong specificity and the ability to provide a global view of the distribution of m^6^A in the transcriptome, and has been widely validated and applied in a variety of plants, including cucurbits. However, MeRIP-seq also has certain limitations, with its resolution typically limited to about 100–200 nucleotides, which poses challenges for the precise identification of m^6^A modification sites and may lead to false positive/negative results due to non-specific binding or incomplete immunoprecipitation [33].

Further exploration of the role of RNA methylation, particularly m^6^A, in the stress response of plant organisms, and how pathogens and viruses evade plant immune defense by influencing methylation patterns, is a current research hotspot. Research has found that m^6^A methylation modification is one of the core mechanisms for plants to resist PNRSV virus. Virus infection alters the m^6^A modification pattern of the host, and m^6^A reader proteins directly inhibit virus accumulation by regulating downstream targets including defense-related genes (such as *PAL*), thereby conferring disease resistance to plants [34]. For example, RNA m^6^A methylation plays a key role in the early immune response of watermelons against cumber green mottle mosaic virus (CGMMV) infection. The study found that disease-resistant watermelons had significantly reduced genome-wide m^6^A levels after CGMMV infection and identified 422 differentially methylated genes, most of which were hypomethylated and associated with upregulation of demethylase gene *ClALKBH4B*. Functional analysis indicated that these genes were enriched in the stress response pathway, and m^6^A methylation levels were generally negatively correlated with gene transcriptional expression. The study revealed for the first time a dynamic map of m^6^A modifications in watermelons in response to viral infection, suggesting that m^6^A demethylation-mediated post-transcriptional regulation is an important component of watermelons’ disease resistance mechanism [31]. Ail et al. found that by using transgenic technology to introduce the direct repeat sequence of the motion protein gene of CGMMV into melons, plants highly resistant to CGMMV were successfully cultivated, and the core of the antiviral mechanism was RNA interference (RNAi). The siRNA produced after transgene transcription effectively targeted and silenced the viral RNA, and the study observed cytosine methylation of the transgenic DNA itself [32].

In addition, m^6^A modification, as a widespread post-transcriptional modification, is widely involved in regulating metabolic processes such as RNA degradation, translation, stability and output, mediating important physiological and pathological processes. These dynamic regulatory mechanisms enable m^6^A modifications to respond to changes in the internal and external environment, thereby influencing gene expression. The CRISPR-Cas13d system developed in recent studies, namely the reprogramming m^5^C modification system (RCMS), provides a new tool for targeting m^5^C methylation [31], which may be extended to the study of m^5^C modifications in virus–host interactions in the future to further clarify their role in biological stress responses. In plants, a variety of RNA methyltransferases (writers), demethylases (erasers), and m^6^A binding proteins (readers) have been identified and studied in depth, which work together to dynamically regulate RNA methylation levels and have a profound impact on gene expression [26]. For example, the TdFIP37 protein is associated with RNA methylation and participates in the maintenance of ionic homeostasis in plants under salt stress, thereby enhancing plant tolerance and viability through complex signaling pathways and regulatory processes [32].

### 3.2. RNA-BisSeq

RNA bisulfite sequencing (RNA-BisSeq) is a powerful technique for detecting m^5^C modifications in RNA at single-nucleotide resolution [33]. Its fundamental principle is that bisulfite treatment converts unmethylated cytosine (C) to uracil (U), while m^5^C in RNA remains unchanged [34]. Subsequent sequencing can identify these C-to-U conversions, enabling precise identification and quantitative analysis of RNA methylation patterns [34,35]. This technique provides quantitative information on RNA methylation levels and can identify m^5^C sites at single-nucleotide resolution, which is crucial for understanding the role of RNA methylation in gene expression regulation and post-transcriptional modification [33,36]. Despite the high-resolution advantage of RNA-BisSeq, this technique also has certain limitations. Its analysis process is relatively complex and requires strict quality control and bioinformatics analysis to accurately distinguish true m^5^C sites from potential sequencing errors. For instance, the bioinformatics workflow for DNA bisulfite sequencing (BS-seq) data analysis typically starts from fastq files, maps sequencing reads to the reference genome using alignment tools (such as bsseeker, bsmap, bismark), then extracts cytosine-related methylation information, and conducts methylation level analysis and single nucleotide polymorphism (SNP) detection [34]. These complex steps are also applicable to RNA-BisSeq. Additionally, due to the harsh chemical conditions during bisulfite treatment, the coverage of BS-seq may be affected compared to other methods such as MeRIP-seq [37,38]. Overall, RNA-BisSeq, as a tool for detecting RNA m^5^C modifications, holds significant value in understanding RNA epigenetics.

m^5^C modification is not only present in DNA but also widely distributed in RNA molecules, and it regulates various biological processes such as RNA metabolism, stability and translation [39,40,41]. In recent years, RNA bisulfite sequencing (RNA-BisSeq) technology has increasingly attracted attention due to its ability to detect m^5^C modifications on a transcriptome-wide scale. For example, the m^5^C-Atlas database included 166,540 m^5^C sites from 13 species, which were identified by five base-resolution epigenetic transcriptome sequencing techniques, further highlighting the wide distribution and importance of m^5^C at the RNA level [39]. In plants, RNA chemical modifications such as m^5^C and m^6^A have been revealed as novel epigenetic markers for regulating gene translation [42]. Although the technique has not yet been found in Cucurbitaceae plants, other studies have shown that plants undergo large-scale transcriptome changes under abiotic stresses such as drought and saline–alkali stress. Transcriptome analyses of *Gossypium purpurascens* (*Gossypium arboreum* L.) under drought, salinity and saline–alkali stress, for example, revealed stress-related gene expression patterns [43]. Regarding the role of RNA m^5^C in adverse stress, the latest research reveals the negative regulatory role of NSUN2-mediated m^5^C methylation in the antiviral innate immune response, suggesting the potential function of m^5^C in organisms’ responses to environmental challenges [40]. In addition, new methods based on XGBoost and SHAP, m^5^C pred-XS, have been developed to predict RNA m^5^C sites in humans, mice, and *A. thaliana*, providing computational tools for more precise identification and study of RNA m^5^C modifications in plants [44]. In conclusion, RNA-BisSeq and its derived RNA bisulfite sequencing technology are key means for studying m^5^C modifications, especially in revealing their role in gene expression regulation and the stress response of organisms. Future studies will continue to use these high-resolution sequencing techniques, combined with bioinformatics methods such as deep learning [45], to more comprehensively analyze the complex mechanisms of m^5^C modification in various biological processes, including plant stress responses.

### 3.3. Nanopore Direct RNA Sequencing

Nanopore direct RNA sequencing (DRS) is an emerging technology that can simultaneously detect multiple RNA modifications, including m^6^A and m^5^C, without the need for chemical transformation or immunoprecipitation. It can preserve the natural RNA structure and provide long-read sequencing data, which greatly revolutionizes transcriptome analysis methods [46]. In the case of Cucurbitaceae crops, although the provided data do not directly relate to specific research cases of nanopore direct RNA sequencing for detecting m^6^A and m^5^C modifications in Cucurbitaceae crops, the potential application and importance can be inferred from related plant studies. For example, in crops such as cotton (*G. purpurascens*), researchers have used PacBio Iso-seq and RNA-seq techniques to analyze their transcriptome dynamics under abiotic stresses such as drought, salinity, and saline–alkali stresses, aiming to gain a deeper understanding of the crop’s response mechanisms to environmental changes [43]. This shows that long-read sequencing techniques have wide applications in analyzing the complex transcriptome of plants.

The long-read nature of nanopore direct RNA sequencing enables more accurate identification of full-length transcripts, which is crucial for understanding alternative splicing and post-transcriptional modifications in complex genomes such as polyploid cucurbitaceae crops [46]. In addition, the technique can directly read RNA sequences, avoiding the bias introduced during reverse transcription and potentially directly detect chemical modifications on RNA, thus providing a more comprehensive perspective for studying the gene expression regulatory network of cucurbit crops at different developmental stages or under environmental stresses such as pest and disease, extreme temperatures, water stress, etc. [46]. By detecting modifications such as m^6^A and m^5^C, scientists can better understand how these modifications affect gene expression, RNA stability, and ultimately agronomic traits such as yield, quality, and stress resistance in cucurbit crops.

## 4. The Application of Epitranscriptomics in Cucurbitaceae Breeding and Genetic Improvement

The practical application of epitranscriptomic knowledge in Cucurbitaceae breeding is moving from theoretical foundations to practical exploration, mainly in two aspects: marker-assisted selection (MAS) and biotechnological breeding. Although these methods are still in their infancy in Cucurbitaceae crops, successful cases in model plants have provided solid proof of concept for their feasibility and great potential.

### 4.1. Identification of RNA Methylation Marks and Their Potential in MAS

MAS is a technique that uses molecular markers closely linked to target traits to efficiently screen breeding materials at early developmental stages, significantly shortening the breeding cycle. Traditional MAS mainly relies on genetic markers at the DNA level, while integrating RNA methylation marks into MAS strategies provides a deeper regulatory dimension for the genetic improvement of Cucurbitaceae crops.

#### 4.1.1. Identification of RNA Methylation Marks

The m^6^A is one of the most common internal modifications of mRNA in eukaryotes, widely present in various RNA types including messenger RNA (mRNA) and non-coding RNA, and influencing gene expression through processes such as RNA splicing, nuclear export, stability, and translation. The dynamic changes of m^6^A modification are closely related to plant growth and development, stress responses, and the formation of specific traits. For instance, in cobalt-induced neurodegeneration studies, the m^6^A demethylase FTO was shown to target TSC1 in an m^6^A-YTHDF2-dependent manner, thereby mediating autophagy dysfunction, revealing the important regulatory role of m^6^A modification in cellular stress responses [44]. In plants, such modifications may also be involved in the response of Cucurbitaceae crops to abiotic stresses (such as drought, salinity, and temperature fluctuations), enhancing the crops’ stress resistance by regulating the expression of related genes [45].

The m^5^C is another important RNA modification, found in various RNAs including tRNA, rRNA, and mRNA [46]. The m^5^C modification also affects RNA structural stability, metabolism, and translation efficiency [46,47]. The m^5^C-Atlas database has collected 166,540 m^5^C sites from 13 species, identified through base-resolution epitranscriptomic analysis techniques, including data from plant species, indicating the wide existence and important functions of m^5^C in RNA [47]. The identification of these RNA methylation marks can reveal potential epigenetic regulatory mechanisms in specific Cucurbitaceae varieties during the formation of specific traits (such as fruit quality, yield, and disease resistance). High-throughput sequencing technologies, such as RNA sequencing (RNA-seq), and enrichment sequencing techniques specific to RNA modifications (such as MeRIP-seq, BS-seq variants, etc.), can identify m^6^A and m^5^C modification sites and their modification levels in Cucurbitaceae crops related to target traits [45]. For example, since the genome sequence of melon (*Cucumis melo* L.) was released, it has greatly accelerated the genetic analysis of candidate genes and QTLs, and the integration of epitranscriptomic data can further enrich this genetic information, providing a more comprehensive view of the gene regulatory network [48].

#### 4.1.2. From Mark Identification to MAS Application

A key step in identification is to map specific RNA methylation sites or their abundance as quantitative traits to the genome through genetic mapping, that is, to identify RNA methylation quantitative trait loci. Once these methQTLs are confirmed to stably co-segregate with key agronomic traits (such as cucumber powdery mildew resistance and sugar accumulation in watermelon fruits), the linked DNA molecular markers (such as single nucleotide polymorphisms, SNPs) can be directly used for early and efficient genotype screening in breeding populations, thereby accelerating the aggregation of favorable alleles [16,49,50].

The success of this strategy has precedents in other species. For instance, DNA marker-assisted selection has been widely applied in rice breeding programs [51] for constructing genetic maps, locating QTLs, and screening germplasm resources. The development of new methods such as marker-assisted reverse breeding (MARB) further demonstrates the great potential of combining epigenetic information with traditional genetic markers [52]. In grape breeding, marker-assisted selection has been widely used for marking QTLs related to disease resistance and fruit maturity [53]. These successful experiences strongly suggest that the application of RNA methylation QTL-associated SNP markers in cucurbit breeding is highly feasible.

### 4.2. Frontiers in Biotechnological Breeding

Biotechnological breeding, by directly manipulating genetic and epigenetic networks, offers unprecedented precision for crop trait improvement [54]. In cucurbit crops, direct intervention in the RNA methylation regulatory circuit itself shows a more global regulatory prospect beyond traditional single-gene editing.

#### 4.2.1. Precise Targeting with CRISPR Gene Editing Technology

The CRISPR/Cas system has demonstrated revolutionary potential in plant gene editing, enabling efficient targeted gene modification [55,56]. Using this technology, the genes of “reader” proteins or “eraser” enzymes that negatively regulate stress responses or fruit development can be precisely knocked out, thereby altering the stability and translation efficiency of a large number of downstream target transcripts and achieving coordinated improvement of complex traits. For example, in wheat, scientists have successfully achieved efficient and heritable genome editing using RNA virus vectors without the need for tissue culture, with mutation frequencies in the next generation of three different wheat lines ranging from 12.9% to 100% [57]. In rapeseed breeding, the BnaIDA gene was edited using CRISPR technology to study its role in flower organ abscission and to rapidly transfer the trait to superior lines [58], providing a technical model for editing similar genes in cucurbits.

#### 4.2.2. Fine-Tuning of “Writer” Enzymes

Another strategy is to positively regulate beneficial RNA methylation modifications. By using tissue-specific or inducible promoters to overexpress specific “writer” enzymes (methyltransferases) at specific developmental stages or in specific tissues, the methylation levels of transcripts that have positive effects on fruit development and stress resistance can be finely tuned, thereby enhancing their stability or translation efficiency and strengthening the target pathways. This strategy can fine-tune the activity of the gene network at the system level, avoiding the potential side effects of drastic changes to a single gene [59]. For example, in radish research, DNA methylation affects energy metabolism and hormone signaling pathways in the formation of F1 hybrid vigor, thereby influencing soluble sugar accumulation and auxin signaling, ultimately leading to the formation of hybrid vigor in main root yield [60]. Although this mainly involves DNA methylation, it provides strong evidence for the role of epigenetic regulation in crop trait improvement.

### 4.3. Concept Validation and Prospects in Model Plants

Although the practical application of the above in cucurbits is still in the early stages of exploration, research in model plants has provided strong principle validation. For instance, studies in Arabidopsis have revealed that AGO3 mainly recruits 24-nt small RNAs to regulate epigenetic silencing [61], which highlights the universality of small RNA-mediated epigenetic regulatory mechanisms in plants. These studies undoubtedly lay a solid theoretical foundation and point out the direction for the targeted improvement of agronomic traits in cucurbit crops by manipulating the RNA methylation regulatory network.

In conclusion, converting epitranscriptomic knowledge into breeding advantages for cucurbit crops is a promising frontier field. By integrating advanced sequencing technologies and bioinformatics to identify key RNA methylation markers and precisely regulating them using biotechnological tools such as gene editing, it is expected to break through the bottlenecks of traditional breeding and ultimately cultivate new varieties of cucurbit crops that are more competitive in the market and adaptable to the environment.

## 5. Key Issues and Limitations of the Study

While significant progress has been made in the field of epitranscriptomics, most research has focused on model plants such as *A. thaliana* and major crops such as rice and tomato. Specific data on Cucurbitaceae plants, particularly in the context of RNA methylation, remain limited. This gap hinders the development of a comprehensive understanding of how RNA modifications specifically regulate the biology of cucurbit plants. Xin et al. [62] provided valuable insights into m^6^A modifications in cucumbers; in watermelons, m^6^A methyltransferase CIMTB was identified as positively regulating drought tolerance, while genome-wide m^6^A demethylation occurred in watermelons during early immunity against CGMMV, and this modification event regulated the expression of stress-related genes [29]. Although important progress has been made in the above research, more extensive and systematic studies are still needed to fully understand the molecular characteristics and biological functions of the epitranscriptomics. Although many studies have identified RNA methylation patterns and their potential roles, the functional validation of these findings remains a challenge. Cucurbitaceae plants lack genetic tools and resources to conduct detailed functional studies such as CRISPR/Cas9-mediated knockout or overexpression experiments. For example, CRISPR/Cas9 technology generated loss-of-function mutants of *CsaMLO1*, *CsaMLO8*, and *CsaMLO11* in powdery mildew-susceptible ADR27 cucumber inbred lines, and obtained and validated the *CsaMLO* mutant, but such studies are still rare in cucumbers [63].

Based on the above analysis, despite significant progress in the field of epigenomtranscriptomics, especially in the study of m^6^A RNA methylation, research in Cucurbitaceae plants in this field still faces multiple limitations that prevent us from fully understanding how m^6^A modification specifically regulates biological processes in cucurbitaceae plants [18,19,20]. Compared with model plants such as *A. thaliana* and major crops such as rice and tomato, there is a serious lack of data on RNA methylation in cucurbiaceae plants [64,65,66,67,68,69]. Most of the existing studies have focused on the growth and development of *A. thaliana* and its response to stress, for example, the dynamic changes in RNA n6-adenine methylation affecting *A. thaliana’s* resistance to Arabidopsis downy mildew [65]. In addition, m^6^A modification in sweet sorghum has shown a significant role in regulating salt tolerance [66]. Dynamic changes in m^6^A methylation during strawberry fruit ripening have also been revealed and are associated with abscisic acid (ABA)-dependent pathways [68]. In contrast, the scarcity of data on cucurbitaceae plants limits a deeper understanding of the epigentranscriptomic functions and mechanisms of these crops. RNA methylation, the most common internal RNA modification in eukaryotes, has a critical impact on RNA stability, translation regulation, and cellular processes and cell fate [16]. However, in the field of plants, especially in cucurbitaceae plants, its biological functions and applications remain to be further explored.

**Figure 1:**
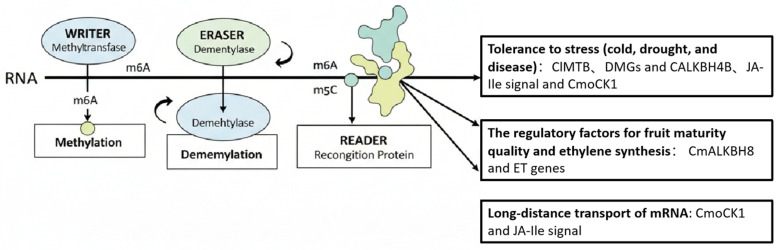
The regulatory role of RNA methylation in the growth, development and stress response of Cucurbitaceae plants. Note: RNA methylation is dynamically regulated by three types of proteins: ‘writers’ (methyltransferases) add methyl marks on RNA as annotations; ‘erasers’ (demethylases) remove these marks; and ‘readers’ (recognition proteins) interpret these marks to determine the fate of RNA, such as its stability, translation or cellular localization. The arrows indicate the “direction of action” or “regulatory flow”. The system shows the process from the enzymatic modification of RNA, to the recognition of the modified sites by recognition proteins, and then to the regulation of specific physiological functions. The arrows pointing from the READER to the three text boxes below indicate that recognition proteins regulate the following biological processes by specifically recognizing RNA methylation modifications: stress tolerance (such as low temperature, drought, and disease), fruit ripening quality, ethylene synthesis, and long-distance transport of mRNA.

RNA methylation, particularly N6-methyladenosine (m^6^A) modification, represents a pivotal post-transcriptional regulatory mechanism that plays a central role in modulating growth, development, and stress responses in cucurbit species (Figure 1). Despite its significance, research on RNA methylation in cucurbits remains constrained by several critical limitations that impede a comprehensive understanding of this intricate regulatory network. Key challenges include limited accumulation of epitranscriptomic data, difficulties in functional validation—especially due to low efficiency in genetic transformation and genome editing—insufficient systematic investigation, and the underdevelopment of molecular tools relative to established model plants [23,24]. Addressing these gaps necessitates the establishment of more efficient transformation systems, the application of cutting-edge sequencing technologies such as nanopore-based direct RNA sequencing to generate high-resolution epitranscriptome profiles, and the integration of multi-omics approaches to fully elucidate the regulatory functions of m^6^A modifications in cucurbit biology.

## 6. Future Research Directions

**Figure 2:**
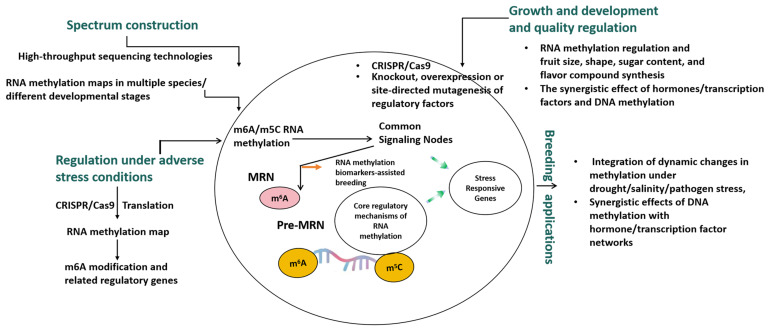
The potential applications of RNA methylation in fruit development, stress response, and breeding and genetic improvement of cucurbit plants.

To fully leverage the application of RNA methylation in fruit development, stress response, breeding and genetic improvement of Cucurbitaceae plants, several key research directions must be given priority (Figure 2). The first is the systematic construction and detailed analysis of the RNA methylation map of Cucurbitaceae plants. High-throughput sequencing technology is needed to systematically map RNA methylation profiles in different cucurbitaceae plants (such as cucumbers, watermelons, melons, etc.) at different developmental stages (fruit development, flowering, vegetative growth) and in different tissues and organs (such as leaves, stems, roots, fruits), especially m^6^A methylation profiles.

The second is the functional validation and genetic manipulation of RNA methylation regulation mechanisms. There is an urgent need to develop efficient genetic transformation and regeneration systems for different Cucurbiaceae species to facilitate the wide application of gene editing tools such as CRISPR/Cas9 for precise knockout, overexpression, or site-directed mutagenesis of specific RNA methylase (writer), demethylase (eraser), and binding protein (reader) genes—to systematically explore the specific functions of these regulatory factors in the growth and development, fruit quality formation, and stress resistance of cucurbitaceae plants.

The third is the analysis of the role of RNA methylation in the growth and development and fruit quality formation of Cucurbitaceae plants. The focus is on how RNA methylation regulates gene expression related to fruit size, shape, sugar content, flavor synthesis, and postharvest storage. Exploring the effects of RNA methylation on flower organ development, flowering period regulation, and sex-determining genes is of great significance for cucurbitaceae crop yield and breeding. Identifying key RNA methylation sites and regulators related to peel color, flesh texture, and nutrient accumulation (such as vitamins and antioxidants) provides new targets for genetic improvement of Cucurbitaceae crop quality.

Fourth, there is a need to systematically study the dynamic changes in the RNA methylation profiles of Cucurbitaceae plants under stress conditions such as drought, saline–alkali, high temperature, low temperature, and pathogen infection, and identify key m^6^A modification events and related regulatory genes. This includes an in-depth analysis of how RNA methylation works in synergy with plant hormone signaling pathways, transcription factor networks, and other epigenetic modifications (such as DNA methylation) to jointly regulate stress responses in Cucurbitaceae plants.

Fifth, the ultimate goal is to apply RNA methylation research results to the breeding and genetic improvement of Cucurbitaceae crops, such as identifying RNA methylation biomarkers closely associated with important agronomic traits (such as high yield, quality, stress resistance) for assisted breeding. We need to explore improving target traits in Cucurbiaceae plants by regulating the expression of RNA methylases or demethylases, such as enhancing crop disease resistance or improving fruit quality by altering m^6^A methylation levels.

Sixth, genotype–epigenotype–phenotype association analysis is needed: By integrating genomics, transcriptomics and epigenomics data, multi-omics integration analysis is carried out to construct the genotype–epigenotype–phenotype association network of Cucurbit plants, thereby providing a more comprehensive understanding of the genetic and epigenetic basis of complex traits. Through these multi-dimensional research directions, RNA methylation studies in cucurbitaceae plants are expected to move from the current preliminary exploration stage to a comprehensive analysis of their molecular characteristics and biological functions, and ultimately be applied to the genetic improvement of cucurbitaceae crops to address the challenges of global food security and climate change.

## 7. Conclusions

Research on the epitranscriptome of cucurbit plants is currently undergoing a pivotal transition from descriptive “mapping” to mechanistic investigation. Recent advances driven by high-throughput sequencing technologies have enabled the preliminary profiling of key RNA modifications—such as m^6^A and m^5^C—across the transcriptome during critical biological processes, including fruit development and stress responses. These profiling efforts have not only uncovered significant associations between dynamic RNA methylation patterns and agronomic traits but also established a foundational framework for guiding future studies. Nevertheless, the field confronts several major challenges: first, incomplete characterization of RNA methylation-related enzyme systems hinders precise genetic manipulation; second, the absence of efficient and specific RNA editing tools limits functional validation, resulting in overreliance on correlative evidence; and third, insufficient integration of multi-omics datasets impedes the establishment of causal links between methylation sites and phenotypic outcomes. To advance the field, a three-pronged strategic approach is essential: (1) the development of high-precision tools for targeted RNA modification to serve as a “molecular scalpel” in functional studies; (2) the systematic integration of multi-omics data to elucidate the regulatory pathways through which RNA modifications influence crop phenotypes; and (3) the implementation of rigorous experimental designs to directly validate the biological functions of key modification sites. Achieving a paradigm shift from passive observation to active intervention will be crucial to transforming RNA methylation from a biologically intriguing phenomenon into a programmable tool for molecular breeding, thereby enabling the development of climate-resilient cucurbit crops and contributing to sustainable agricultural innovation.

## Figures and Tables

**Table 1:** The regulatory role of RNA methylation in the growth, development and stress response of cucurbit plants.

Species	Published/Available Data on RNA Methylation	Regulatory Factor	Physiological Processes	Reference
Cucumbers and pumpkins (*Cucumis sativus* L.) and (*Cucurbita moschata* D.)	m^6^A modification is related to RNA mobility and has breeding potential	Genome-wide mRNA (including m^5^C and m^6^A modification sites)	RNA mobility, crop breeding	[25]
Oriental melon (*Cucumis melo* var. *Makuwa*)	m^6^A methylation positively regulates fruit ripening and ethylene synthesis.	*CmALKBH8*, ethylene genes	Fruit ripening, ethylene biosynthesis	[28]
Watermelon (*Citrullus lanatus*)	m^6^A methylation enhances drought resistance through ClMTB.	CIMTB	Drought stress response	[29]
Cucumbers and Pumpkin (*Cucumis sativus* L.) and (*Cucurbita moschata* D.)	m^6^A and m^5^C directly regulate the long-distance mobility of *CmoCK1* mRNA at low temperatures.	*CmoCK1*, JA-Ile signal	Cold tolerance, long-distance mRNA transport, stress response	[30]
Watermelon (*Citrullus lanatus*)	Decreased m^6^A levels activate early immunity.	DMGs, *CALKBH4B*	Early virus immunity	[31]
Melon (*Cucumis melo* L.)	Transgenic activation of RNA silencing mechanism confers antiviral ability. siRNA, methylated	siRNA, methylated DNA	RNA silencing-mediated high plant disease resistance	[32]

## Data Availability

No new data were created or analyzed in this study.
